# The Hospital Almoner

**Published:** 1924-06

**Authors:** M. W. Edminson

**Affiliations:** Almoner, Royal London Ophthalmic Hospital


### THE HOSPITAL ALMONER.

### WHAT SHE IS AND WHAT SHE DOES.

By MISS M. W. EDMINSON, ALMONER, ROYAL LONDON OPHTHALMIC HOSPITAL.

It is impossible in any short space to give a comprehensive survey of the work of a Hospital Almoner, but the following article may briefly suggest one or two sides of the work of the social service department.

### Small Beginnings.

Social work in the hospitals began in a very small way. We are proud to remember that it first started at the Royal Free Hospital in London, through the inspiration of Sir Charles Lock, and it is interesting to note how, very shortly afterwards, Dr. Cabot conceived the same idea in America and founded there a social service department on almost similar lines. When Miss Cummins, the Head Almoner of St. Thomas's Hospital, started in the Casualty Department it was with the definite idea of acting as a link between the patients and the outside world. The need was very great, because it was impossible for the members of the surgical, nursing or administrative staff to devote time to after-care work. Some of them, it is true, out of the goodness of their hearts, would spend their time, when they should have been seeking relaxation and rest, in endless services to the patients. One sister was known, after a day of most strenuous charge of a surgical ward, to go out in all weathers to visit a child whose wound needed dressing at home, and there was a doctor who regularly (unknown to almost everyone) took milk every day to one of his tuberculosis patients who otherwise would have gone without.

### Where the Almoner is Wanted.

Here is where the Almoner is wanted; and if she is to do justice to the work she needs a thorough knowledge not only of the lives and difficulties of the patients, but also of all those many institutions, associations, and wheels within wheels which go to make up the social life of the nation, someone, too, who has studied the efforts of workers in the past and has learnt from their failures and successes. The child's wound required daily dressing; the Nursing Association was willing to do it, but the bond was missing. There lay the duty of the Almoner—to put the nurse and the patient in touch with one another and to give the surgeon's recommendation for the treatment. For a long time it had been realised that a bottle of medicine was not a panacea for all evils, and that it was not enough to tell an anæmic girl to live in the open air nor the wage-earner of a family to stop work. How could they? They wanted someone who through the help of a wider education and an extended imagination could sort out and solve the problems and make the apparently impossible things possible.

### The Story of "Annie."

One of the first duties of an Almoner is therefore to act as a link between the patient and the outside world, and in carrying on the work there is always a second and most important factor to remember—the interdependence of doctor, sister and Almoner. Ill-health, physical and neurological troubles are so often caused by adverse social and economic conditions that to attempt to nurse a patient back to life only to let him return to the conditions which have caused the trouble is, at the very least, a short-sighted policy. There was a girl at one of the London hospitals who was sent to a convalescent home four times in two years, each of the periods being for three months. She was a thin, pale girl "all on wires," and the physician reported that she had early thyroid disease and must be sent away to the sea with complete freedom from worry and anxiety of any kind. There seemed on the face of things no reason for worry; her home was apparently a happy one, her people fairly well off, and she was earning good money from a firm that proved to be consideration personified. Her place was kept open, she had some sick pay, and it seemed as though there was nothing for her to do but get well. Yet after two months in August she broke down again in October, and though she was well enough to start work in January, in May another breakdown was imminent. This time she was sent to another home. It was then discovered that Annie had been engaged for eight years, and it had ended unhappily—just as she was getting over the trouble the news of the man's marriage upset her again.

### Gaining the Patient's Confidence.

The doctor in the light of this discovery advised work, and she bravely struggled to start off again, and for some months made a splendid fight and held her own. Then once more she came to the hospital, and this time she told the Almoner her real trouble. There were two causes of a very real sort. Her father, a most affectionate and very respectable man, had terrible drinking fits. The distress of seeing him in this condition and the ghastly uncertainty each evening was more than the daughter's weakened health could bear. And beyond this the man who had treated her so badly was working every day in the same factory, and she was faced with the ever-present ache of the old wound. Annie agreed to one plan, and that was to go and stay with her brother whenever her father was "unwell," and the fact that she had spoken freely of the other cause helped her very considerably to face things bravely.

### Convalescent Homes.

In many provincial towns, as, for example, in Sheffield, sufficient money has been raised to obtain various beds at different convalescent homes, so that hospitals can continue the treatment in splended country air and under excellent conditions. That, of course, is exactly what is needed. But many of the convalescent homes are limited, either by the scarcity of beds or because they are not equipped to take special cases. No convalescent home will take a child suffering from acute eye trouble requiring treatment; but what the ophthalmic surgeon wants is a convalescent home and a staff who can give ophthalmic treatment.

